# Tailoring Silicon Nitride Surface Chemistry for Facilitating Odontogenic Differentiation of Rat Dental Pulp Cells

**DOI:** 10.3390/ijms222313130

**Published:** 2021-12-04

**Authors:** Yanan Gong, Yoshitomo Honda, Tetsuya Adachi, Elia Marin, Kazushi Yoshikawa, Giuseppe Pezzotti, Kazuyo Yamamoto

**Affiliations:** 1 Department of Operative Dentistry, Osaka Dental University, 8-1 Kuzuhahanazonocho, Hirakata 573-1121, Japan; gong-y@cc.osaka-dent.ac.jp (Y.G.); kazushi@cc.osaka-dent.ac.jp (K.Y.); yamamoto@cc.osaka-dent.ac.jp (K.Y.); 2 Department of Oral Anatomy, Osaka Dental University, 8-1 Kuzuhahanazonocho, Hirakata 573-1121, Japan; 3 Department of Dental Medicine, Graduate School of Medical Science, Kyoto Prefectural University of Medicine, Kajiicho, Kawaramachi-Hirokoji, Kamigyo-ku, Kyoto 602-8566, Japan; t-adachi@koto.kpu-m.ac.jp (T.A.); elia-marin@kit.ac.jp (E.M.); pezzotti@kit.ac.jp (G.P.); 4 Department of Ceramic Physics Laboratory, Kyoto Institute of Technology, Sakyo-ku, Matsugasaki, Kyoto 606-8585, Japan

**Keywords:** dental pulp cells, odontogenic differentiation, silicon nitride, surface wettability, cell adhesion

## Abstract

Silicon nitride (Si_3_N_4_) can facilitate bone formation; hence, it is used as a biomaterial in orthopedics. Nevertheless, its usability for dentistry is unexplored. The aim of the present study was to investigate the effect of Si_3_N_4_ granules for the proliferation and odontogenic differentiation of rat dental pulp cells (rDPCs). Four different types of Si_3_N_4_ granules were prepared, which underwent different treatments to form pristine as-synthesized Si_3_N_4_, chemically treated Si_3_N_4_, thermally treated Si_3_N_4_, and Si_3_N_4_ sintered with 3 wt.% yttrium oxide (Y_2_O_3_). rDPCs were cultured on or around the Si_3_N_4_ granular beds. Compared with the other three types of Si_3_N_4_ granules, the sintered Si_3_N_4_ granules significantly promoted cellular attachment, upregulated the expression of odontogenic marker genes (*Dentin Matrix Acidic Phosphoprotein 1* and *Dentin Sialophosphoprotein*) in the early phase, and enhanced the formation of mineralization nodules. Furthermore, the water contact angle of sintered Si_3_N_4_ was also greatly increased to 40°. These results suggest that the sintering process for Si_3_N_4_ with Y_2_O_3_ positively altered the surface properties of pristine as-synthesized Si_3_N_4_ granules, thereby facilitating the odontogenic differentiation of rDPCs. Thus, the introduction of a sintering treatment for Si_3_N_4_ granules is likely to facilitate their use in the clinical application of dentistry.

## 1. Introduction

Dental caries are the most prevalent form of chronic disease in both adults and children worldwide [1,2,3]. Promoting the formation of restorative dentin in decayed areas is thus considered a common clinical treatment method [4]. Pulp tissue contributes to the production of dental hard tissue, including restorative dentin in response to physiological and pathologic stimuli [5,6]. Dental pulp cells are derived from mesenchymal stem cells and have multiple differentiation potential [6,7]; when suitably stimulated, pulp cells can differentiate into odontoblasts and secrete dentin matrix [8,9,10,11]. Thus, the vitality of pulp tissue and formation of dentin bridges determine the success of pulp capping treatment [12,13]. 

Currently, calcium hydroxide is well accepted clinically, owing to its ability to promote the formation of dentin bridges and calcific barriers [14,15,16]. However, calcium hydroxide does not adhere to dentin and dissolves over time [12], causing the formation of tunnel defects in dentin bridges [17] and leading to the inflammation and necrosis of the pulp tissue [18,19]. Therefore, the development of new pulp-capping agents is essential.

Previous studies have reported that silicon nitride (Si_3_N_4_) is a non-cytotoxic [20,21] and biocompatible (with the ISO 10993) [22] ceramic material that improves in vitro osteoblast differentiation [20,23] and apatite formation [24]. Si_3_N_4_ implantation in animal femurs [25,26] was found to promote bone formation around the implant [27,28]. Si_3_N_4_ implants are FDA-approved and already in clinical use [22,29] as vertebral body fixation implants. The microspectroscopic examinations of a short-term retrieval spinal implant demonstrated that Si_3_N_4_ possesses a peculiar surface chemistry that greatly accelerates bone repair in vivo [30]. However, the influence of Si_3_N_4_ on dental pulp cells and its optimal surface conditions remain unexplored to date.

To explore the novel application of Si_3_N_4_ as a dental material, such as pulp covering material, the aim of the present study was to compare the effects of Si_3_N_4_ granules with different surface stoichiometries on the viability and odontogenic differentiation of rat dental pulp cells (rDPCs).

## 2. Results

### 2.1. Characterization of Si_3_N_4_ after Different Treatments

Four types of Si_3_N_4_ granules were prepared: pristine as-synthesized Si_3_N_4_ (P-Si_3_N_4_), treated in glacial acetic acidic (A-Si_3_N_4_), thermally oxidized at 200 °C (T-Si_3_N_4_), and high-temperature sintered Si_3_N_4_ (S-Si_3_N_4_) with the addition of 3 wt.% Y_2_O_3_ (Table 1). 

**Figure 1:**
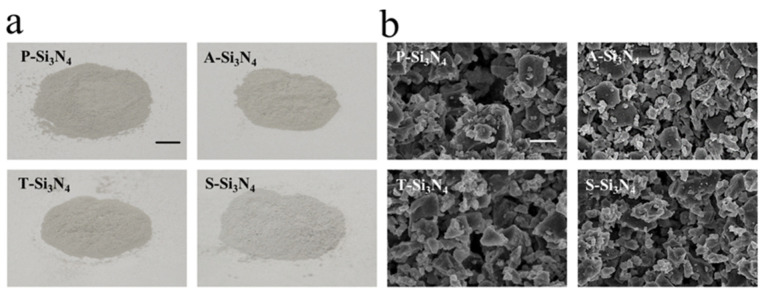
Characterization of Si_3_N_4_. (**a**) Macroscopic images of Si_3_N_4_. Scale bar = 5 mm. (**b**) Field emission scanning electron microscopy (FE-SEM) images of Si_3_N_4_. Scale bar = 10 µm. (P-Si_3_N_4_: Pristine Si_3_N_4_, A-Si_3_N_4_: Acid-treated Si_3_N_4_, T-Si_3_N_4_: Thermal-treated Si_3_N_4_, S-Si_3_N_4_: Sintered Si_3_N_4_ with 3 wt.% Y_2_O_3_).

The macroscopic and Field emission scanning electron microscopic (FE-SEM) images of four different Si_3_N_4_ powders are shown in Figure 1. The thermal and acid treatment did not cause any noticeable change in the color of the material (Figure 1a). FE-SEM observations at higher magnifications ×10.0 k (Figure 1b) showed that the four powders have a comparable average grain size and similar morphology; they include only a few large particles with a diameter in the order of tens of microns, and a dispersion of smaller micro- and trans-micrometric particles. 

**Figure 2:**
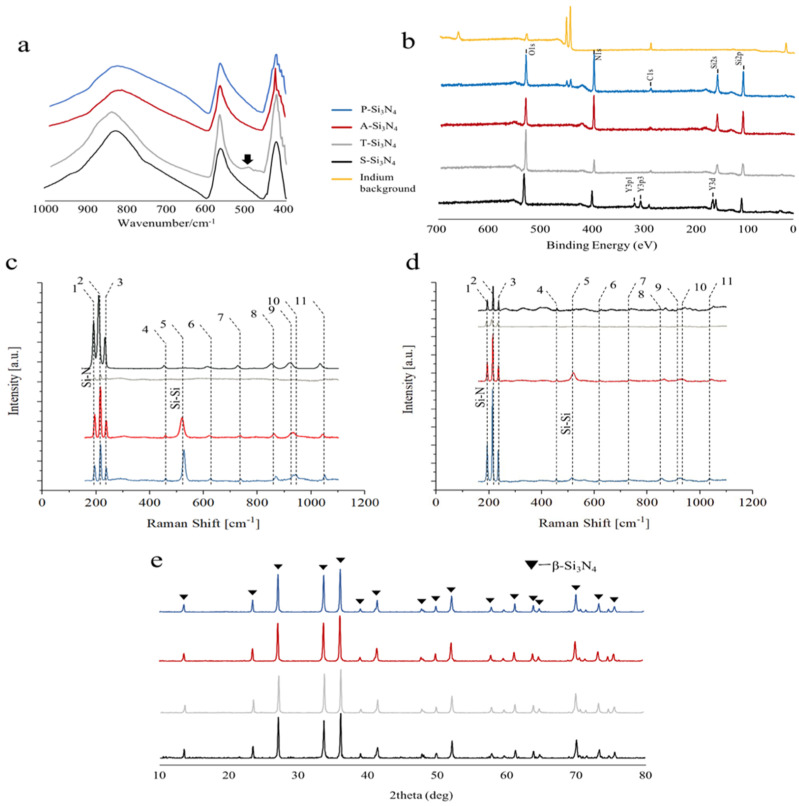
(**a**) Attenuated total reflectance−Fourier transform infrared spectroscopy (ATR−FTIR) spectra of Si_3_N_4_. Arrows: Si-O bonds. (**b**) X−ray photoelectron spectroscopy (XPS) spectra showing the elements contained in the manufactured Si_3_N_4_. (**c**) Raman spectroscopy results with green excitation sources. (**d**) Raman spectroscopy results with near infra-red excitation sources. (**e**) X−ray diffraction (XRD) pattern of Si_3_N_4_ crystals (P-Si_3_N_4_: Pristine Si_3_N_4_, A-Si_3_N_4_: Acid-treated Si_3_N_4_, T-Si_3_N_4_: Thermal-treated Si_3_N_4_, S-Si_3_N_4_: Sintered Si_3_N_4_ with 3 wt.% Y_2_O_3_).

When investigated by Attenuated total reflection–Fourier transform infrared (ATR-FTIR) spectroscopy (Figure 2a), the four powders produced similar results; the main difference was a peak at 493 cm^−1^, related to Si-O bonds, which was only visible for the T-Si_3_N_4_ sample. This result suggests that at the thermal treatment at 200 °C, the amount and depth of the silica outer layer spontaneously formed on the particles in humid environments [24].

X-ray photoelectron spectroscopy (XPS) analyses (Figure 2b) confirmed the presence of high amounts of oxygen on the T-Si_3_N_4_ sample when compared to both the A-Si_3_N_4_ and the P-Si_3_N_4_ samples. The A-Si_3_N_4_ showed a surface with a higher content of nitrogen, whereas yttrium, as expected, could only be detected in the S-Si_3_N_4_ powder sample.

The Raman spectroscopy results with green and near infra-red excitation sources are shown in Figure 2c,d, respectively, whereas the main assignments from band deconvolution are shown in Table 2. The main bands are consistent with the results previously reported in the literature [31], with the exception of a relatively strong band at about 520 cm^−1^ that can be assigned to residual, unreacted amorphous, or micro-crystalline silicon [32]. While the acid post-treatment seems to increase the intensity of the silicon band, the oxidation treatment reduces all band intensities due to the formation of a surface layer of silica, with no active bands in the analyzed spectral window. For the sample containing Y_2_O_3_, the absence of the Raman band at 520 cm^−1^ suggests a complete reaction during sintering. When compared to the reference spectra (P-Si_3_N_4_) under green light excitation, the triplet of bands in the region between 180 and 250 cm^−1^ appear to be broader. This phenomenon was previously associated with the formation of Si–Y–O–N compounds [23] that were speculated to contribute to bone formation in vitro. 

The powder X-ray diffraction (XRD) patterns of all samples represented the Si_3_N_4_ β-phase as the predominant phase (Figure 2e). The phase transition evidenced by both ATR-FTIR and XPS could not be detected by XRD, meaning that it was limited to the outer surface of the ceramic particles.

### 2.2. Characterization of Rat Dental Pulp Cells

**Figure 3:**
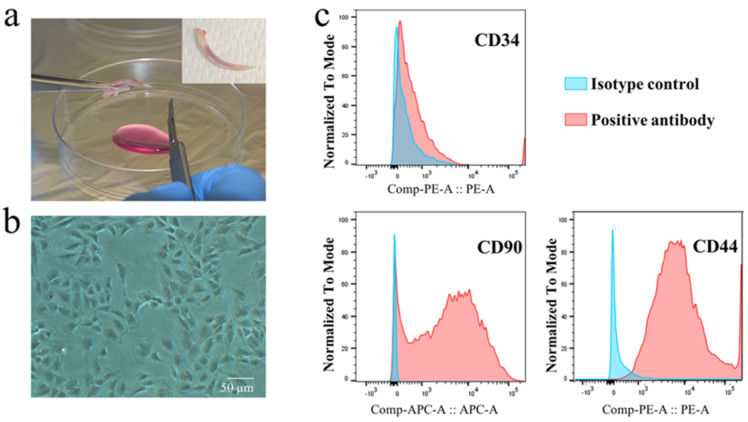
Characterization of primary rat dental pulp cells (rDPCs). (**a**) Mandibular incisors were extracted from rats for isolating rDPCs. (**b**) Microscopic examination showed a fibroblast−like morphology in fourth passage rDPCs. Scale bar = 50 µm. (**c**) Immunophenotype assay by flow cytometric analysis.

The rDPCs were isolated from the mandibular incisor of rats (Figure 3a,b). The expression of cell surface markers CD90, CD34, and CD44 was assessed to identify the cell type of rDPCs. The presence or absence of these markers form the criteria for identifying stem cells. The isolated cells presented a high expression of CD90 and CD44 (the well-known marker of dental pulp stem cells [9,35]) and low expression of CD34 (a primitive hematopoietic progenitor and endothelial cell marker) (Figure 3c), suggesting that the prepared rDPCs are likely heterogeneous cells, including dental pulp stem cells.

### 2.3. Proliferation of rDPCs Cultured with Si_3_N_4_

**Figure 4:**
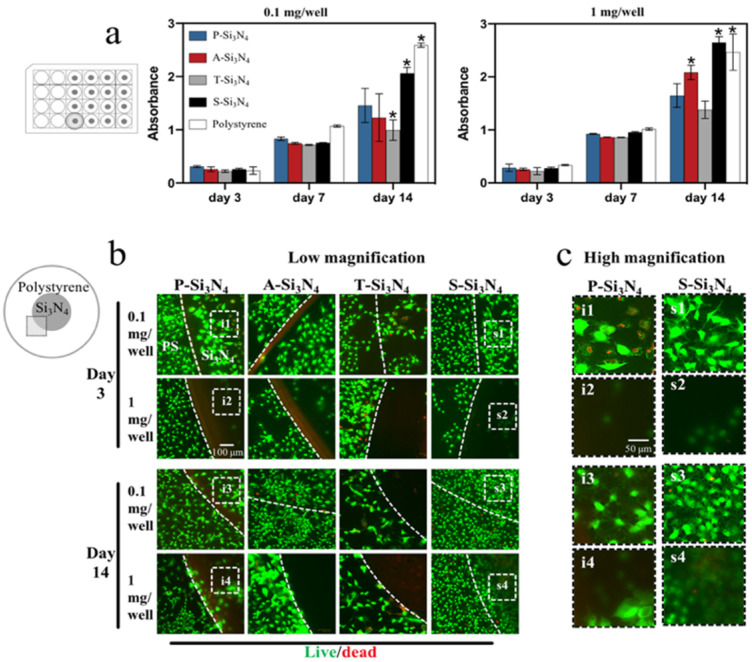
Evaluation of cytotoxicity in vitro. (**a**) Cells incubated with 0.1 and 1 mg/well of Si_3_N_4_ for different times (3, 7, and 14 days) were analyzed by Cell Counting Kit-8 assay. Data are presented as the mean ± standard deviation (SD) (*n* = 3). The differences of mean values among the test group were evaluated at one-way analysis of variance and Tukey’s multiple comparisons test. * *p* < 0.05: vs. the P-Si_3_N_4_ group. (**b**) Live/dead viability staining showing the cell activity of the surface with and without Si_3_N_4_ coating. Green: live cells; red: dead cells. Scale bar = 100 µm. (**c**) High magnification views of P-Si_3_N_4_ and S-Si_3_N_4_. Scale bar = 50 µm. (P-Si_3_N_4_: Pristine Si_3_N_4_, A-Si_3_N_4_: Acid-treated Si_3_N_4_, T-Si_3_N_4_: Thermal-treated Si_3_N_4_, S-Si_3_N_4_: Sintered Si_3_N_4_ with 3 wt.% Y_2_O_3_).

Cell viability on and around the Si_3_N_4_s was analyzed using wells containing different doses of the ceramics at the center (Figure 4). Fourteen days after cell culture, the total number of cells in the well coated with S-Si_3_N_4_ showed more significant proliferation compared to that with other Si_3_N_4_s (Figure 4a). Using live or dead staining at the edge of Si_3_N_4_ and the polystyrene surface (gray square in Figure 4b), we found that the number of cells on the P-, A-, or T-Si_3_N_4_ decreased with time compared with that on the polystyrene surface (around Si_3_N_4_), whereas there was a negligible difference with the cells on the S-Si_3_N_4_ at day 14 (Figure 4b). 

### 2.4. Real-Time Reverse Transcription Quantitative Polymerase Chain Reaction (Real-Time qPCR) Measurement

**Figure 5:**
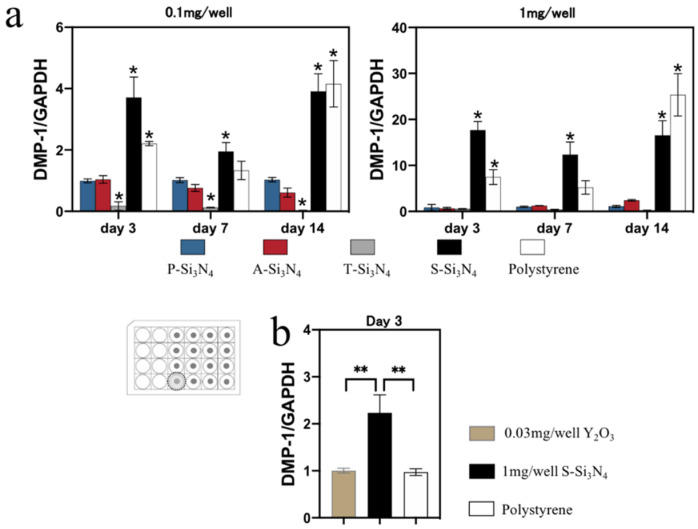
Real-time qPCR to detect the expression of odontoblast-specific genes. (**a**) *Dentin Matrix Acidic Phosphoprotein (DMP-1)* expression at 3, 7, and 14 days on plates coated with P-, A-, T-, S-Si_3_N_4_. (**b**) *DMP-1* expression at 3 day on plates coated with 1 mg/well of S-Si_3_N_4_ (3 wt.% Y_2_O_3_ added) and Y_2_O_3_ (0.03 mg/well). Data are presented as the mean ± SD (*n* = 3). The differences of mean values among the test group were evaluated at one-way analysis of variance and Tukey’s multiple comparisons test. (**a**) * *p* < 0.05: vs. the P-Si_3_N_4_ group; (**b**) ** *p* < 0.01: comparison among all groups. (P-Si_3_N_4_: Pristine Si_3_N_4_, A-Si_3_N_4_: Acid-treated Si_3_N_4_, T-Si_3_N_4_: Thermal-treated Si_3_N_4_, S-Si_3_N_4_: Sintered Si_3_N_4_ with 3 wt.% Y_2_O_3_).

After treatment with an odontogenic medium, the cells in the S-Si_3_N_4_-coated wells expressed a higher level of *Dentin Matrix Acidic Phosphoprotein 1* (*DMP-1*) (Figure 5) and *dentin sialophosphoprotein* (*DSPP*) (Supplementary Figure S1) from day three compared to the cells on other Si_3_N_4_s or on polystyrene. 

### 2.5. Mineralization Nodules

**Figure 6:**
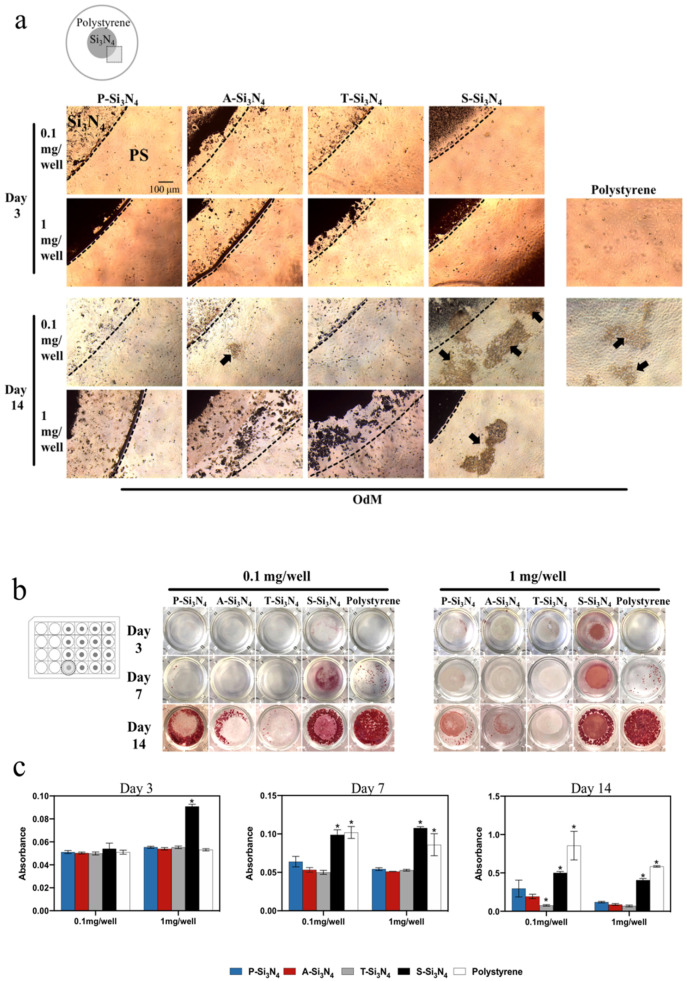
(**a**) Microscopic observation of rDPCs cultured under odontogenic conditions with or without Si_3_N_4_ for 3 and 14 days. Arrows: Mineralization nodules, Scale bar = 100 µm. (**b**) Effects of Si_3_N_4_ on calcium deposition examined by alizarin red S staining. (**c**) Histogram of alizarin red S staining quantification. Data are presented as the mean ± SD (*n* = 3). The differences of mean values among the test group were calculated at one-way analysis of variance and Tukey’s multiple comparisons test. * *p* < 0.05: vs. the P-Si_3_N_4_ group. (P-Si_3_N_4_: Pristine Si_3_N_4_, A-Si_3_N_4_: Acid-treated Si_3_N_4_, T-Si_3_N_4_: Thermal-treated Si_3_N_4_, S-Si_3_N_4_: Sintered Si_3_N_4_ with 3 wt.% Y_2_O_3_, OdM: odontogenic differentiation medium).

Mineralized nodule-like structures could be found in the wells with S-Si_3_N_4_, but not in the wells of other Si_3_N_4_s except for the 0.1 mg/well A-Si_3_N_4_ (arrows in Figure 6a). The wells treated with S-Si_3_N_4_ showed increased alizarin red staining at earlier time points compared to other Si_3_N_4_-coated wells (Figure 6b,c). Conversely, there was little red staining on and around T-Si_3_N_4_ subjected with thermal treatment even after 14 days of cell culture, which was weaker than that observed with P-Si_3_N_4_.

### 2.6. Raman Characterization

**Figure 7:**
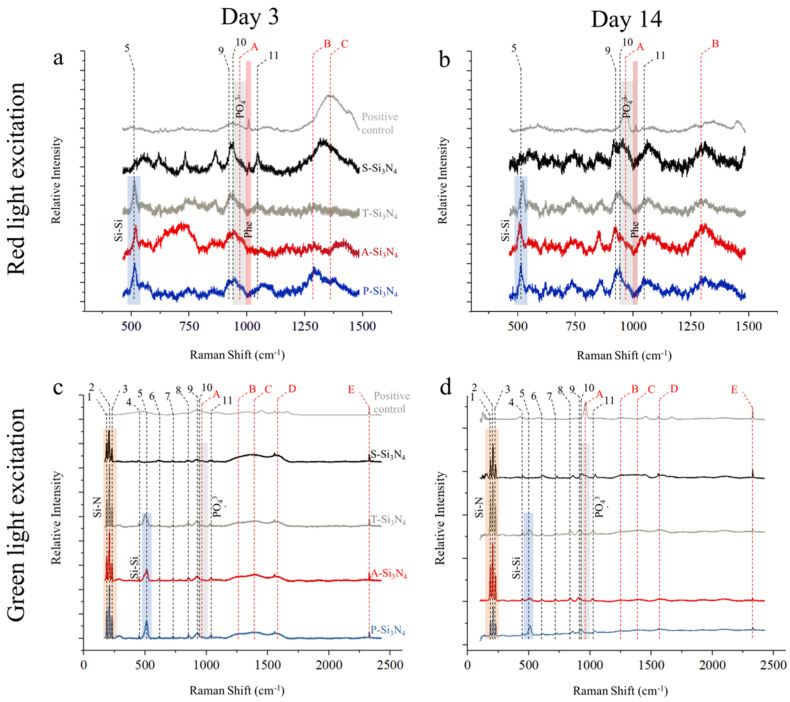
Raman analyses performed after 3 and 14 days of in vitro culture. Raman detection with a red laser after 3 (**a**) or 14 (**b**) days of incubation. Raman detection with a green laser after 3 (**c**) or 14 (**d**) days of incubation. Representative data are presented from three replicate experiments. Numbering and characters are the same as those used in Table 2.

Raman analyses performed after 3 and 14 days of in vitro culture are shown in Figure 7. On day 3, with the exception of S-Si_3_N_4_ and the positive control, spectra obtained with red light excitation (Figure 7a) showed a prominent silicon band at about 520 cm^−1^, as previously observed in Figure 2c,d. Two bands related to Si_3_N_4_ were clearly visible at about 900 cm^−1^, followed by a shoulder band (A), which was caused by PO_4_^3-^ vibrations in bone apatite. At higher Raman shifts, a band related to collagen amide II (B) could be observed, particularly on the P-Si_3_N_4_ sample, whereas the last band at about 1380 cm^−1^ was derived from the glass substrate. The band at about 1004 cm^−1^ and highlighted in red is related to the presence of phenylalanine and was only visible with the positive control and with the S-Si_3_N_4_ sample. On day 14 (Figure 7b), the relative intensity of the bands (A) and (B) increased for all samples, particularly for the positive control and for S-Si_3_N_4_. The phenylalanine signal is barely visible in S-Si_3_N_4_, but is clearly observed in the positive control.

Results obtained under green light excitation are less sensitive to organic material, as confirmed by the spectra obtained after 3 days of in vitro culture (Figure 7c), which strongly resemble those in Figure 2c. The band at about 520 cm^−1^ appears to have a lower relative intensity when compared with the relatively pristine powders, whereas two distinguished bands, (C) and (D), can be attributed to organic matter burning under the power of the laser beam. The results at 14 days (Figure 7d) show the presence of the hydroxyapatite band (A) on both the positive control and the S-Si_3_N_4_ sample.

### 2.7. The Effect of Y_2_O_3_ in S-Si_3_N_4_ on Odontogenic Differentiation

*DMP-1* expression levels of the rDPCs in S-Si_3_N_4_-coated wells were higher than those cells in wells coated with Y_2_O_3_ alone (Figure 5b). The cells in both wells showed a different expression pattern. 

### 2.8. Surface Wettability of the Si_3_N_4_ Surface

**Figure 8:**
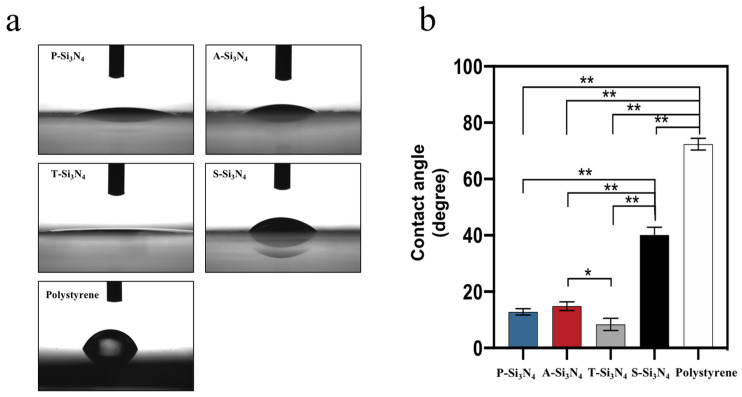
(**a**) Macroscopic images of water absorption at the silicon nitride-coated surface. (**b**) Measurement of the contact angle on the surface of Si_3_N_4_. Data shown are the means ± SD (*n* = 3). The differences of mean values among the test groups were evaluated by one-way analysis of variance and Tukey’s multiple comparisons test. * *p* < 0.05, ** *p* < 0.01. (P-Si_3_N_4_: Pristine Si_3_N_4_, A-Si_3_N_4_: Acid-treated Si_3_N_4_, T-Si_3_N_4_: Thermal-treated Si_3_N_4_, S-Si_3_N_4_: Sintered Si_3_N_4_ with 3 wt.% Y_2_O_3_).

The surface wettability of materials strongly affects cell adhesion [36]. The contacts angle of the S-Si_3_N_4_ surface was significantly higher than that of the surfaces of other Si_3_N_4_s (Figure 8).

## 3. Discussion

Our data show that the Si_3_N_4_ granules prepared with three different treatments (acid treatment, thermal treatment, and sintered treatment) showed markedly altered surface chemistry compared to the pristine Si_3_N_4_ granules. The powder bed made of S-Si_3_N_4_ granules, which underwent a sintering process with the addition of Y_2_O_3_, showed higher water contact angles compared to the other Si_3_N_4_s. Coincident with this change, S-Si_3_N_4_ facilitated the increased proliferation and odontogenic differentiation of rDPCs compared to that with other Si_3_N_4_s. 

Four Si_3_N_4_s were selectively prepared because of following reasons: the original idea was to compare the as-synthesized (P-Si_3_N_4_) powder with the sintered (S-Si_3_N_4_) one; the former being highly pure, was expected to induce the strongest pH buffering effect and thus the highest amount of ammonia eluted, while the latter containing the Y_2_O_3_ additive as a sintering aid [37] and being thus alloyed by it, was expected to have a milder pH buffering effect and a lower elution of ammonia. Tailoring the pH buffering effect is considered to be key in boosting up cell metabolism without damaging the cells, as ammonia elution beyond a given (unknown so far and different for different types of cells) concentration threshold could be hard for the cells to metabolize. An alternative route to control elution was tried by tuning only the outer surface chemistry: the A-Si_3_N_4_ sample was treated with acetic acid because there are proofs that treatments in concentrated acetic acid can be used to remove (by esterification) the Si-OH silanol groups that form on the surface of Si_3_N_4_ [38]. The silanol groups are then restored in aqueous environments, making the surface of the A-Si_3_N_4_ powders more reactive in the initial stages of hydrolysis; The T-Si_3_N_4_ sample was obtained by treating the powder at a relatively low temperature (200 °C) in order to produce a thin SiO_2_ layer on the outer surface, with the ultimate goal of reducing the reactivity of the powder surface [39] without affecting the bulk of the material.

The expression of *DMP-1* was significantly increased in the early phase of differentiation (day 3 and day 7) in the S-Si_3_N_4_ group compared to that in the other Si_3_N_4_s (Figure 5). *DMP-1* expresses during odontogenic differentiation [40]. DMP-1 can nucleate hydroxyapatite formation by binding calcium ions [41]. However, *DMP-1* expression was significantly reduced in mature odontoblasts in vitro [42]. Although we could not elucidate the terminal differentiation of rDPCs due to the limited culture period (14 days), the S-Si_3_N_4_ granular bed facilitated the early differentiation of rDPCs, suggesting that Si_3_N_4_ prepared at optimal conditions must have a latent ability to initiate the odontogenic differentiation of rDPCs.

As mentioned above, along with being an early marker of odontogenic differentiation, DMP-1 and DSPP are known to be associated with mineralization [43,44]. Alizarin red staining and Raman spectroscopy are the conventional tools to identify the calcium and phosphate in mineralization [45,46,47]. In our data, the red staining on S-Si_3_N_4_ was the strongest compared to that with the other Si_3_N_4_ groups (Figure 6b). Raman spectroscopy showed significant phosphate spectra on the surface of the S-Si_3_N_4_ at 960 cm^−1^ (Figure 7). These results support the evidence that DMP-1 and DSPP protein expression, possibly induced by S-Si_3_N_4_, may partially contribute to early mineralization.

Despite the paucity of information regarding the ability of yttrium to induce odontogenic differentiation, the elements in S-Si_3_N_4_ were likely to modulate the odontogenic differentiation of rDPCs. However, our data indicate that the expression of *DMP-1* in rDPCs treated with S-Si_3_N_4_ is inconsistent with that induced by Y_2_O_3_ alone (Figure 5b), suggesting that odontogenic differentiation induced by S-Si_3_N_4_ was mainly due to other stimuli rather by yttrium alone, at least for the used doses. The cell adhesion and surface wettability of materials are known to be key regulators of cell differentiation [48,49]. Furthermore, the surface wettability of materials strongly affects cell adhesion [36]. In our results, S-Si_3_N_4_ showed a higher water contact angle (Figure 8), which was the closest to the optimum angle for cell adhesion [36]. In fact, this finding was consistent with the live or dead staining data in Figure 4b and odontogenic differentiation in Figure 5, Figure 6 and Figure 7. Based on these results, the sintering process promoted the odontogenic differentiation of rat pulp cells on S-Si_3_N_4_, partially through the alteration of its surface properties.

Previous studies have evaluated the local and systemic biological response of Si_3_N_4_ in cell cultures and animal models [25,26]. Si_3_N_4_ has been shown to be biocompatible in vivo in experiments on sheep [26] and rabbit femurs [25]. Additionally, KUSA-A1 cells [23], SaOS-2 cells [24], and MG63 cells [20] have been found to be effective at in vitro osteogenesis. Most of these findings confer precious insights for bone formation and osteoblastic differentiation, whereas there has been no direct information regarding odontogenic differentiation from pulp cells. To the best of our knowledge, this is the first study using cultured rDPCs isolated from rat dental pulp tissue on and around Si_3_N_4_ with different surface properties, indicating that Si_3_N_4_s latently have the ability to alter rDPC activity.

Thermally treated T-Si_3_N_4_ induced significantly attenuated *DMP-1* expression and mineralized nodule formation compared to that with intact Si_3_N_4_ (P-Si_3_N_4_). This observation suggests a role for nitrogen species leaving the surface of Si_3_N_4_ upon hydrolysis, as suggested previously by one of the authors [50]. Further detailed examination will be necessary to find a better process (concentration, temperature, or additives) for optimizing each Si_3_N_4_ surface effect in dentistry. For example, it is still unclear whether or not the concurrent presence of Y_2_O_3_ plays an essential role. In such a case, the optimal sintering temperature or doses of Y_2_O_3_ remain to be determined. While a complete description of the mechanisms underlying the superior behavior of S-Si_3_N_4_ in activating rDPCs is missing, the byproducts of hydrolytic reactions of Si_3_N_4_, such as silanols and nitrogen species including nitride oxide [51], might be associated with the observed proliferation and differentiation of rDPCs. Moreover, the effect of S-Si_3_N_4_ in dentin formation in vivo remains to be tested. However, our results clearly indicate that different treatments could be effective in altering the surface properties of Si_3_N_4_ to better increase its bioactivity and to enhance odontogenic differentiation. The present investigation thus provides new insights into the development of novel dental materials based on Si_3_N_4_ bioceramics.

## 4. Materials and Methods

### 4.1. Preparation of Si_3_N_4_ Granules

Four types of Si_3_N_4_ granules were prepared: pristine as-synthesized Si_3_N_4_, treated for 72 h in glacial acetic acidic ≥ 99%, thermally oxidized for 72 h at 200 °C, and 1600 °C high-temperature sintered Si_3_N_4_ with the addition of 3 wt.% Y_2_O_3_ (Table 1). To ensure the homogeneity of Si_3_N_4_ granules, these four types of granules were ground separately and filtered to obtain granules with sizes ranging from 20 to 75 µm, which were then used for the experiments. 

### 4.2. Characterizations of Si_3_N_4_

FE-SEM images were obtained using the S-4800 FE-SEM system (Hitachi, Tokyo, Japan). All samples were coated with OsO_4_ using a Vacuum Device (Ibaraki, Japan). The crystalline phases of Si_3_N_4_ determined by powder XRD (LabX XRD-6000, SHIMADZU Corporation. Kyoto, Japan), and the chemical structures were characterized by ATR-FTIR spectroscopy (IRAffinity-1S; Shimadzu Corporation, Kyoto, Japan). Chemical composition and chemical bonding were determined by XPS (PHI X-tool; ΦULVAC-PHI, Inc., Kanagawa, Japan) and Raman Spectroscopy (RAMAN touch, Nanophoton, Osaka, Japan). 

### 4.3. Coating of Cell Culture Plates

Two concentrations of Si_3_N_4_ suspensions were added to the center of 24-well cell culture plates (0.1 and 1 mg/well). After drying at 50 °C for 24 h, the plates were used for cell cultures. All plates were pre-sterilized by exposure to UV light.

### 4.4. Primary Culture of rDPCs

rDPCs were isolated from the incisor dental pulp tissues of 5-week-old male Wistar-ST rats (Shimizu Laboratory Supplies, Kyoto, Japan). All animal experiments were approved by the Animal Research Committee of Osaka Dental University and performed strictly according to the guidelines (Approval No. 21-02012; approval date: 23 March 2021). The obtained tissues were incubated with collagenase type I (3 mg/mL: Wako Pure Chemical Industries, Osaka, Japan) at 37 °C for 40 min. The fluid containing the cells was centrifuged for 3 min (1000× *g*). The cells were cultured in a Minimum Essential Medium Eagle-Alpha Modification (Nacalai Tesque, Kyoto, Japan), containing 20% fetal bovine serum and 1% penicillin-streptomycin solution (designated as culture medium), at 37 °C in a humidified atmosphere with 5% CO_2_. The fourth passage cells were used for this study. To characterize the immunophenotype of rDPCs, the antigen normally expressed on stem cells (APC anti-CD90, PE anti-CD44) and hemopoietic stem cells (PE anti-CD34) were selected. The cells were analyzed by flow cytometry using the FACSVerse^TM^ system (BD, Franklin Lakes, NJ, USA). 

### 4.5. Cell Proliferation Assay

rDPCs were seeded on 24-well plates coated with or without Si_3_N_4_ (0.1 and 1 mg/well) at 4.0 × 10^4^ cells/well in culture medium. After 3, 7, and 14 days in culture, the Cell Counting Kit-8 (Dojindo Laboratories, Kumamoto, Japan) was used to assess cell proliferation, and absorbance was measured at 450 nm using a plate reader (SpectraMax M5; Molecular Devices, San Jose, CA, USA). The LIVE/DEAD^TM^ Viability/Cytotoxicity Kit was used to assess the state of cellular activity on surfaces with or without Si_3_N_4_ coating. The results of live/dead fluorescence staining were obtained using the ZOE Fluorescent Cell Imager (Bio-Rad Laboratories, Hercules, CA, USA).

### 4.6. Real-Time qPCR Assay

rDPCs were cultured in odontogenic differentiation media (OdM), which were prepared by culture medium supplemented with 10 mM glycerol 2-phosphate, 10 (or 100) nM dexamethasone, and 50 (or 155) µM L-ascorbic acid 2-phosphate (FUJIFILM Wako Pure Chemical Corporation, Osaka, Japan). The RNeasy Mini Kit (QIAGEN, Hilden, Germany) was used to extract total RNA from rDPCs. cDNA was synthesized using the SuperScript™ VILO™ cDNA Synthesis Kit (Invitrogen, Thermo Fisher Scientific Inc., Waltham, MA, USA). The mRNA levels of the odontoblast-related DMP-1 and DSPP were investigated using the Step One^TM^ Plus RT-PCR System (ThermoFisher Scientific, Waltham, MA, USA). Glyceraldehyde 3-phosphate dehydrogenase (GAPDH) was regarded as the internal control, and the ΔΔCT method was used for quantifying gene expression. The accession numbers of the TaqMan gene expression assay PCR system are as follows: DMP-1, Rn01450122_m1; DSPP, Rn02132391_s1; GAPDH, Rn01775763_g1.

### 4.7. Mineralization Assay

Mineralization nodules were observed by alizarin red S (Sigma-Aldrich, St Louis, MO, USA) staining. rDPCs were cultured in OdM for 3, 7, and 14 days. The medium was aspirated, and the wells were washed with phosphate buffered saline at the respective time points. Cells were fixed with 4% paraformaldehyde, and stained with alizarin red solution for 30 min. To quantify the mineralization nodules, the stain was extracted with 10% formic acid, and the absorbance of the resulting solution was measured at 415 nm using the plate reader (SpectraMax M5).

### 4.8. Raman Experiment

Each Si_3_N_4_ or the formation of mineralized nodules on the surface of Si_3_N_4_ granules were evaluated with a laser Raman microscope (RAMAN touch, Nanophoton, Osaka, Japan) using a 100×.objective lens with a 532.07 nm wavelength green laser and a 785.13 nm wavelength NIR laser. Sample positioning was achieved using a x-y stage controlled with step motors, while an auto-focus function on the Z-axis was used to optimize the signal output. Both the green and NIR laser operated at a nominal power of 200 mW; however, to prevent burning, the power output was reduced by using a neutral-density filter.

At cell culture experiments, cells were cultured on glass plates (MatTek, Ashland, MA, USA) containing 1 mg/well of Si_3_N_4_ granules for 3 and 14 days using OdM. The group without any Si_3_N_4_ coating, under the same incubation conditions, was used as the positive control.

### 4.9. Surface Wettability

The water contact angle of the Si_3_N_4_-coated surface of the wells was measured separately using contact angle meter LSE-ME (NiCK Corporation, Saitama, Japan) and i2win software.

### 4.10. Statistical Analysis

Statistical analysis was performed using GraphPad Prism 8 (GraphPad Software Inc., San Diego, CA, USA). All experiments were replicated at least two times. The differences in mean values among the test groups were evaluated using one-way analysis of variance and Tukey’s multiple comparisons test. A value of *p* < 0.05 was considered to indicate significant differences.

## 5. Conclusions

In the present study, we found that sintered granules with the addition of Y_2_O_3_ possessed altered hydrophilicity compared to pristine as-synthesized Si_3_N_4_ granules. This resulted in increased pulp cell adhesion, which may promote the odontogenic differentiation of rDPCs in vitro. Conversely, Si_3_N_4_ granules subjected to thermal treatment at 200 °C in air significantly attenuated the proliferation and differentiation of rDPCs and the formation of mineralized nodules. These results suggest that sintering pre-treatments of Si_3_N_4_ could be optimized to alter its surface properties, offering an optimal bioactive surface for this bioceramic in dental applications. In summary, this study demonstrates that upon suitable pre-treatment, Si_3_N_4_ could be considered a novel candidate in the clinical application of dentistry.

## Figures and Tables

**Table 1:** Preparation of silicon nitrides.

Type of Si_3_N_4_	Abbreviations	Treatment
Pristine-Si_3_N_4_	P-Si_3_N_4_	As-synthesized
Acid-Si_3_N_4_	A-Si_3_N_4_	Acetic acid, 72 h
Thermal-Si_3_N_4_	T-Si_3_N_4_	200 °C, 72 h
Sintered-Si_3_N_4_	S-Si_3_N_4_	Sintered at 1600 °C, Y_2_O_3_ (3 wt.%) added

**Table 2:** The main assignments from band deconvolution.

No	Position (Red)	Position (Green)	Assignation	References
1	193	186	E_2g_	[31]
2	215	210	A_g_	[31]
3	235	230	E_1g_	[31]
4	455	455	E_2g_	[31]
5	520	520	Si-Si (crystalline)	[32]
6	615	621	E_2g_	[31]
7	725	730	A_g_	[31]
8	855	865	E_1g_	[31]
9	910	930	E_2g_	[31]
10	930	945	A_g_	[31]
11	1035	1050	E_g_	[31]
A	935	960	PO_4_^3-^ *v1*	[33]
B	1260	1255	Amide II	[33]
C	1365	1360	D band	[34]
D	-	1680	G band	[34]
E	-	2310	Led light emission	

## Data Availability

Not applicable.

## Supplementary Materials

The following are available online at https://www.mdpi.com/article/10.3390/ijms222313130/s1.
