# Supplementary material for: Tailoring Silicon Nitride Surface Chemistry for Facilitating Odontogenic Differentiation of Rat Dental Pulp Cells
Source: Int J Mol Sci. 2021 Dec 4;22(23):13130. doi: 10.3390/ijms222313130 (PMC8658470; doi:10.3390/ijms222313130)
Supplement: Supplementary file 1 [file ijms-22-13130-s001.zip › ijms-1464544-supplementary.pdf]

## Supplementary materials

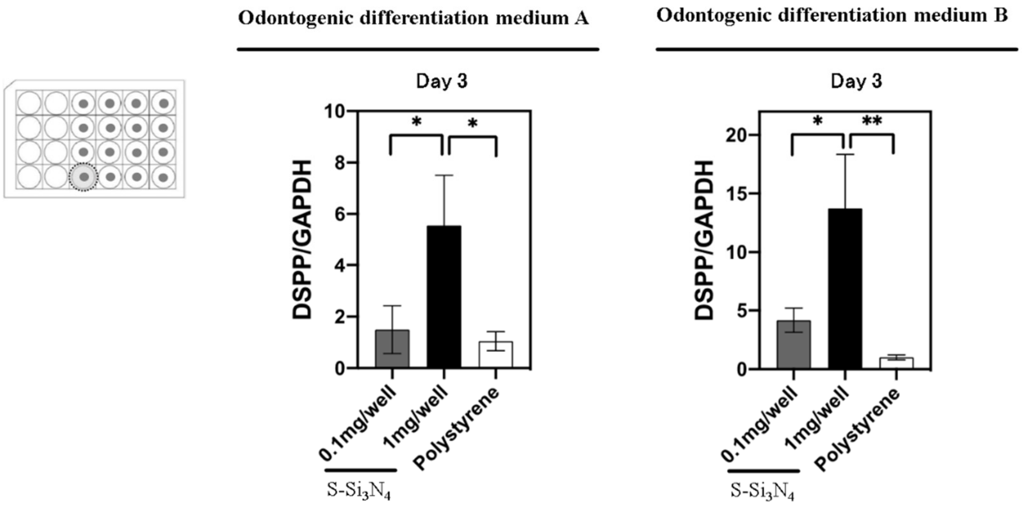


**Supplementary Figure S1.** Real-time qPCR to detect the expression of odontoblast-specific gene, *DSPP*, at day 3 on plates coated with S-$Si_3N_4$. Data are presented as the mean ± standard deviation (SD) (n = 3, one-way analysis of variance and Tukey's multiple comparisons test). *$p < 0.05$, **$p < 0.01$: comparison among all groups. Osteogenic differentiation medium A: culture medium with 10 mM glycerol 2-phosphate, 10 nM dexamethasone, and 155 μM L-ascorbic acid 2-phosphate; Osteogenic differentiation medium B: culture medium with 10 mM glycerol 2-phosphate, 100 nM dexamethasone, and 50 μM L-ascorbic acid 2-phosphate. S- $Si_3N_4$: Sintered $Si_3N_4$ with 3wt.% $Y_2O_3$.
